# A Deep Sequence Learning Framework for Action Recognition in Small-Scale Depth Video Dataset

**DOI:** 10.3390/s22186841

**Published:** 2022-09-09

**Authors:** Mohammad Farhad Bulbul, Amin Ullah, Hazrat Ali, Daijin Kim

**Affiliations:** 1 Department of Computer Science and Engineering, Pohang University of Science and Technology (POSTECH), 77 Cheongam, Pohang 37673, Korea; 2 Department of Mathematics, Jashore University of Science and Technology, Jashore 7408, Bangladesh; 3 CORIS Institute, Oregon State University, Corvallis, OR 97331, USA; 4 College of Science and Engineering, Hamad Bin Khalifa University, Qatar Foundation, Doha P.O. Box 34110, Qatar

**Keywords:** 3D action recognition, depth map sequence, CNN, transfer learning, bi-directional LSTM, RNN, attention

## Abstract

Depth video sequence-based deep models for recognizing human actions are scarce compared to RGB and skeleton video sequences-based models. This scarcity limits the research advancements based on depth data, as training deep models with small-scale data is challenging. In this work, we propose a sequence classification deep model using depth video data for scenarios when the video data are limited. Unlike summarizing the frame contents of each frame into a single class, our method can directly classify a depth video, i.e., a sequence of depth frames. Firstly, the proposed system transforms an input depth video into three sequences of multi-view temporal motion frames. Together with the three temporal motion sequences, the input depth frame sequence offers a four-stream representation of the input depth action video. Next, the DenseNet121 architecture is employed along with ImageNet pre-trained weights to extract the discriminating frame-level action features of depth and temporal motion frames. The extracted four sets of feature vectors about frames of four streams are fed into four bi-directional (BLSTM) networks. The temporal features are further analyzed through multi-head self-attention (MHSA) to capture multi-view sequence correlations. Finally, the concatenated genre of their outputs is processed through dense layers to classify the input depth video. The experimental results on two small-scale benchmark depth datasets, MSRAction3D and DHA, demonstrate that the proposed framework is efficacious even for insufficient training samples and superior to the existing depth data-based action recognition methods.

## 1. Introduction

The research on Human Action Recognition (HAR) has attracted the widespread attention of the computer vision research community during the last decade. Indeed, the vast spectrum of applications of HAR in daily life has stimulated researchers to be dedicated to the issue significantly. Because of the developments in HAR automated systems, the machine intelligence penetration has increased in applications such as human–machine and human–object interaction, content-based video summarizing, education and learning, healthcare systems, entertainment systems, safety and surveillance systems, and sports video analysis [1,2,3,4,5,6]. However, the earlier attempts to recognize actions mostly relied on RGB videos [7,8,9,10]. These methods may result in promising performance on HAR in a limited number of cases; however, RGB data-based recognition approaches have some serious limitations, as they are susceptible to illumination variation, occlusions, and cluttered backgrounds.

To address the limitations of RGB data-based methods, the imaging technology society has invented the depth sensor (e.g., Kinect sensor). The depth sensor works as a multi-modal sensor and thus simultaneously delivers the depth and RGB videos of a scene. However, the depth video-based approaches are illumination, color, and texture invariant [11]. Moreover, depth video preserves the 3D structure of an object accurately, which helps the system to alleviate the intra-class variation and the cluttered background noise issues [11]. Thus, computer vision researchers have shown an increasing interest in approaching the task of action recognition by employing depth data features. Furthermore, the skeleton action sequences can be easily obtained from the depth action sequences. Hence, the skeletal action features have also been utilized in building a recognition system, such as [12,13].

Previous Work: In recent years, deep learning models and convolutional neural networks (CNNs) [14] have been used massively for recognizing image contents. CNNs extract dominant and discriminating object characteristics automatically, and hence, they became popular for extracting features as compared to handcrafted descriptors. Being inspired by the performance of CNNs in image classification tasks, many researchers have applied them in action video classification challenges. However, those action classification works were mostly developed on RGB and skeleton data. For example, deep models as reported in [4,6,13,15,16,17,18,19,20,21,22,23,24,25,26,27,28] are developed on RGB and skeleton action data. There are only a small number of deep models based on the depth video streams only such as those illustrated in [29,30,31,32,33,34,35,36,37]. However, the existing depth databases, excluding the recent NTU-RGB-D databases [38,39], are not large enough for training deep models. In a few studies, the depth data have been complemented with other data modalities such as RGB and skeleton data to develop multi-modal/hybrid deep models [40,41,42].

Many hand-designed methods [43,44,45,46,47,48,49,50,51,52,53,54,55,56,57,58,59,60,61,62] were proposed by researchers before the work on deep learning methods for depth action recognition. These methods usually involve many operations that require researchers to carry out careful feature engineering and tuning [63]. In addition, hand-crafted features and methods are always shallow and dataset dependent [32]. On the other hand, deep learning methods reduce the need for feature engineering. As a result, researchers have attempted to work with deep learning in action recognition from depth videos. For example, in [64], 2D CNNs and 3D CNNs were proposed for depth action recognition. To preserve the temporal information of depth action sequences in DMMs-based action representation, the DMM pyramid was constructed and fed into the 2D CNN as input. The DMM cube was used as input of 3D CNN. The 2D CNN model on DMM pyramid provided comparable and considerable results. Wang et al. [37] proposed a deep model to address the action recognition task on a small-scale training dataset. They utilized three weighted hierarchical depth motion maps (WHDMMs) and the three-streams convolutional neural networks to build their architecture. In fact, the introduction of weights in WHDMMs helps to preserve the temporal order of motion segments to reduce the inter-class similarity problem. The three WHDMMs were constructed from the projection of depth videos onto three-dimensional space. They were converted to pseudo-color versions and fed into three individual CNNs (trained on ImageNets) for training the deep model. The fusion of classification outcomes of the three deep networks was treated as the final classification outcome.

A four-channel CNN pipeline was proposed by [34], where three channels adopted the three types of depth motion maps obtained from depth data, and the fourth channel received the RGB data-based motion history images as input. In the method discussed by [32], an action was described through dynamic depth images, dynamic depth normal images and dynamic depth motion normal images. The three descriptions of the action were treated as input of three-stream CNN architecture for action classification. As a different approach, the depth action representation was considered through the RGB data features directly by domain adaptation in [33]. Wu et al. [11] constructed the hierarchical dynamic depth projected difference images for three projection images and fed them into three uniform CNN. In [65], depth videos were projected on the 3D space with multiple viewpoints, and multi-view dynamic images were constructed. These dynamic images were fed into a novel CNN for feature learning. The fully connected layers of CNN were different for different dynamic images. Finally, with the deep features, the actions were classified using the linear SVM after dimension reduction with PCA.

Keceli et al. [66] fused spatial and temporal deep features obtained from the 2D CNN (pre-trained) and 3D CNN, respectively. The 2D and 3D representations of depth action videos were prepared prior to pass them to the 2D and 3D deep CNN architectures. However, the Relieff algorithm [67] was applied for selecting the most potential features from the fused version. Finally, the SVM classifier was used for the action classification with the selecting features. Li et al. [68] derived a set of three motion images against each input video and then employed the local ternary pattern encoded images for representing action with rich texture information and less noise. The encoded images were passed to a CNN for the action classification. Indeed, the threshold value choosing of the local ternary pattern is a bit difficult. However, Wu et al. [69] represented a depth action video through dynamic image sequences. Then, a channel was proposed to highlight the most dominant channels in CNNs. In addition, a spatial–temporal interest points (STIPs) attention model was proposed to extract the discriminating motion regions from the dynamic images. In their work, an LSTM model was utilized for gaining the temporal dependencies and for accomplishing the classification task. Recently, unlike extracting features from dynamic images, Tasnim et.al. [29] proposed a method extracting features from raw depth images. They used a 3D CNN model for the key-frame-based feature extraction and classification tasks. The key frames were selected by structural similarity index measure (SSIM) and correlation coefficient measure (CCM) metrics for removing the redundant frames as well as preserving more informative frames. In [30], the spatiotemporal action features were extracted using a 3D fully convolutional neural network from raw depth images. The same network also allows action classification. The method was evaluated on the large-scale dataset, i.e., the NTU RGB+D [38] dataset. The statistical features and 1D CNN features were fused for developing an action recognition model from depth action sequences [31]. Multi-channel CNN and a classifier ensemble were utilized in [35]. The method described in [36] employed the 2DCNN and 1DCNN consecutively as pre-processing tools to extract statistical features from depth frames. Those features were fused with the Dynamic Time Wrapping (DTW) algorithm-based statistical features. For the feature classification task, a classifier ensemble was determined from 1000 sets of classifiers. This method seems very complicated since, in the pre-processing stage, it trained a separate CNN model for each action class.

Since the development of deep models based on depth action data only is hard due to limited training data, researchers have been motivated to incorporate other data modalities with depth data. For example, deep learning-based action recognition was presented in [70] using depth sequences and skeleton joint information combined. A 3D CNN structure was used to learn the spatiotemporal features from depth sequences, and then joint-vector features were computed for each sequence. Finally, the SVM classification results of the two types of features were fused for action recognition. In work [71], the fuzzy weighted multi-resolution DMMs (FWMDMMs) were constructed by using the fuzzy weight functions on depth videos. The FWMDMMs were fed into a convolution neural network deep model for the compact representation of actions. In addition to the motion features, the appearance features were also extracted from the RGB and depth data through the pre-trained AlexNet network. Multiple feature fusion techniques were used to obtain the most discriminating features. The multi-class SVM was implemented to classify actions. In [72], the authors used the RGB data features with the depth data features to propose a deep framework. The framework inputs four streams such as Dynamic image, DMM-front, DMM-side and DMM-top. The first one was obtained from the RGB data, and the remaining three streams were generated from the depth data. Those four streams were passed to four pre-trained VGG networks for feature extraction and training. The obtained four classification scores from the classification layers of the four networks were fused using a weighted product model. In [40], the authors proposed a two-stream 3D deep model using depth and RGB action data. The depth residual dynamic image sequence and pose estimation map sequence were calculated simultaneously from depth and RGB modalities of an action. For describing and obtaining the classification score of the action with two modalities, 3D CNN was employed on two individual data streams. The action class was determined by the fusion of the classification scores provided by the 3D CNN on the two data streams. In [42], an action classification algorithm was developed using RGB, depth, and skeleton data modalities. On one hand, the RGB and depth videos were passed to 3D CNN for extraction. On the other hand, 3D CNN and LSTM were employed to capture action features from the skeleton data. Three sets of extracted features were fed into three SVM to obtain probability scores. Two evolutionary algorithms were used to fuse those scores and to output the class label of the input video.

Research Motivation and Key Contribution: The aforementioned depth data-based existing deep models (except the model in [69]) are not able to classify a depth frame sequence directly using sequence classification models such as LSTM, bi-directional LSTM (BLSTM), GRU, bi-directional GRU, or attention models. However, there are many approaches based on the RGB and skeleton data that are capable of classifying a frame sequence automatically using those models [73,74,75,76,77]. The size of the available depth training dataset is the key barrier to developing a depth video-dependent sequence learning deep model. Currently, only two large-scale datasets, NTU RGB+D [38] and NTU RGB+D120 [39], are available with a large number of depth training samples for the sequence learning framework development. Otherwise, the existing depth action video datasets have insufficient depth training videos for the task. Up-to-date, depth data-based deep models (except the model in [69] and 3D CNN-based models) are mainly predicting an action class for an input action video-based on an image classification strategy instead of a direct sequence classification strategy [29,30,31,32,33,34,35,36,37]. Actually, a large number of video sequences is needed in the training stage to develop a promising sequence classification framework using the deep sequence modeling algorithm, which is not available in depth datasets except in the two above datasets. However, there is a need to make progress on deep models trained on small-scale depth datasets. In this work, we propose a deep model for small-scale depth datasets for directly classifying a depth frame sequence. Being inspired by the excellent performance of CNN in automatic feature extraction and representation in depth, RGB, and skeleton action recognition methods [13,17,20,21,24,25,26,27,28,30,31,32,73], we also utilize a pre-trained 2D CNN named DenseNet121 [78], trained on an ImageNet image dataset [79], for capturing dominant features to represent independent action frames. With the extracted features, the combination of the BLSTM [80] and the multi-head self-attention (MHSA) [81] mechanisms are considered to build a sequence classification model. To the best of our knowledge, no previous work has utilized BLSTM and MHSA individually or jointly with deep features to propose such a sequence classification model in-depth video classification problem. We evaluate our method on two public depth action datasets. The performance evaluation shows that our method achieves superiority over many state-of-the-art methods.

Our research contributions are highlighted as follows:

- Learned patterns extraction using deep models with a small-scale dataset is very challenging. To address this issue, we employed a unified framework of BLSTM and MHSA to achieve better sequence-based action recognition in depth videos.
- We propose a single depth video representation through four data streams to boost the depth action representation. The four data streams have a single depth frame sequence and three temporal motion frame sequences. The depth frame sequence is the original input sequence, and the other three sequences are derived from the original one. The other three motion sequences preserve the spatiotemporal motion cues of the front, side, and top flank performers.
- Frame level features extraction is an essential step for sequence-based decisions for action recogntion. We employ a pre-trained 2D CNN model with a transfer learning strategy for robust depth features representations.
- The sequence classification model is developed with the one-to-one integration of BLSTM and MHSA layers. A set of optimal parameters for the BLSTM-MHSA combination is determined, providing the key support for the performance improvement of the proposed method.
- BLSTM-MHSA correlation features are encoded with fully connected layers with a features dropout strategy to achieve model generalization for the unseen test set.
- An ablation study is also provided for different 2D CNN models and the number of data streams for robust action classification.
- The proposed method is assessed in terms of two public datasets, MSRAction3D [82] and DHA [83], and our results are compared with other state-of-the-art methods. In summary, our method exhibits superiority over the recent (published on 20 April 2022) state-of-the-art 3D CNN-based recognition method [29] by 1.9% for MSRAction3D and by 2.3% for DHA. In contrast to the 3D CNN model, our approach involves fewer video frames in each sequence and fewer trainable parameters.

The rest of this paper is oriented as follows: The proposed framework is illustrated in detail in Section 2. Experimental evaluation is discussed in Section 3. Finally, Section 4 concludes the paper.

## 2. Proposed System

This section discusses our proposed system in terms of several subsections where each sub-section clarifies individual component comprehensively.

### 2.1. Four-Stream Action Representation

**Figure 1:**
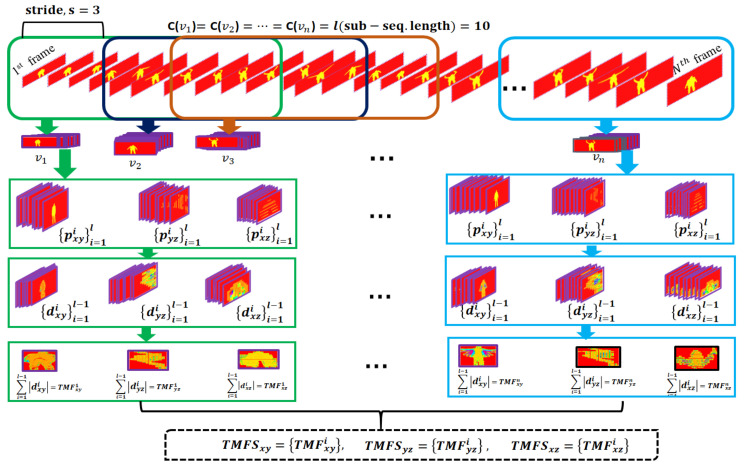
A step-by-step example of temporal motion frame sequence generation.

We hypothesize that the action representation through the raw depth frame-based features as well as motion frame-based features ia more discriminating. Thus, a raw depth video or depth frame sequence (DFS) of length *N* is mapped to produce sequences of multi-view temporal motion frames. To compute those sequences, an overlapping sliding window of size *l* frames is employed on a DFS. The sliding window moves over the DFS with a stride *s* and crops *m* number of chunks/sub-sequences ${\left\{v_{j}\right\}}_{v=1}^{m}$ (where *j* represents the index of a chunk). All the chunks are basically subsets of DFS such as $DFS=\cup_{j=1}^{m}v_{j}$ and maintain a uniform number of frames, i.e., $C\left(v_{1}\right)=C\left(v_{2}\right)=\ldots =C\left(v_{m}\right)=l\in Z^{+}$ (C means number of frames/length of chunk). All the frames of $v_{1}$ chunks are projected onto a 3D coordinate space. The motion frame gathers all the motion segments of the front flanks of frames in $v_{1}$. The consecutive differences among all its projection frames relevant to the xy-plane are calculated and added to generate a motion frame. Another two motion frames are computed corresponding to the yz-plane and xz-plane projection frames. The motion frames about the yz-plane and xz-plane accumulate motion segments of the side and top flanks of frames in $v_{1}$. Consequently, there are three motion frames generated from the chunk $v_{1}$ with respect to the three 2D planes. Note that the motion frames for $v_{1}$ about the planes are obtained based on a sliding window of a fixed stride, i.e., temporal chunks. Thus, the gained motion frames are referred to as temporal motion frames, which are in a single set as $\{\left(TMF_{xy}^{1}\right)$, $\left(TMF_{yz}^{1}\right)$, $\left(TMF_{xz}^{1})\right\}$. Similarly, a single set of three motion frames are computed for every remaining chunks $v_{2},v_{3},\ldots ,v_{m}$ such as $\left\{TMF_{xy}^{2},TMF_{yz}^{2},TMF_{xz}^{2}\right\},\ldots ,\left\{TMF_{xy}^{m},TMF_{yz}^{m},TMF_{xz}^{m}\right\}$. Mathematically, the motion frame generation of any chunk $v_{j}$ about the three planes could be expressed as

(1) $$TMF_{xy}=\sum_{i=1}^{l-1}|d_{xy}^{i}|,$$

(2) $$TMF_{yz}=\sum_{i=1}^{l-1}|d_{yz}^{i}|,$$

(3) $$TMF_{xz}=\sum_{i=1}^{l-1}|d_{xz}^{i}|,$$

where $d_{xy}^{i}=\left(p_{xy}^{i+1}-p_{xy}^{i}\right)$, $d_{yz}^{i}=\left(p_{yz}^{i+1}-p_{yz}^{i}\right)$ and $d_{xz}^{i}=\left(p_{xz}^{i+1}-p_{xz}^{i}\right)$ are distances between successive projections ${\left\{p_{xy}^{i}\right\}}_{i=1}^{l}$, ${\left\{p_{yz}^{i}\right\}}_{i=1}^{l}$, and ${\left\{p_{xz}^{i}\right\}}_{i=1}^{l}$ on the three planes of depth frames of any chunk $v_{j\in \{1,2,\cdots ,m\}}$. However, three sequences of motion frames are obtained by organizing all the temporal motion frames about the xy-plane, yz-plane and xz-plane. The three temporal motion sequences (TMFS) are $TMFS_{xy}=\left\{TMF_{xy}^{1},TMF_{xy}^{2},\cdots ,TMF_{xy}^{m}\right\}$, $TMFS_{yz}=\left\{TMF_{yz}^{1},TMF_{yz}^{2},\cdots ,TMF_{yz}^{m}\right\}$, and $TMFS_{xz}=\left\{TMF_{xz}^{1},TMF_{xz}^{2},\cdots ,TMF_{xz}^{m}\right\}$ with respect to the xy-plane, yz-plane and xz-plane, respectively. Indeed, a single depth action video is transformed into three temporal motion sequences (TMFS), and those sequences capture the spatiotemporal motion information of an entire action. In this work, the data transformation is taken with a stride s=3 and the length of chunk l=10 empirically. An example of such a transformation is shown in Figure 1.

A single depth frame sequence is transformed into three different sequences $TMFS_{xy}=\left\{TMF_{xy}^{1},TMF_{xy}^{2},\cdots ,TMF_{xy}^{m}\right\}$, $TMFS_{yz}=\left\{TMF_{yz}^{1},TMF_{yz}^{2},\cdots ,TMF_{yz}^{m}\right\}$, and $TMFS_{xz}=\left\{TMF_{xz}^{1},TMF_{xz}^{2},\cdots ,TMF_{xz}^{m}\right\}$. The integration of the original depth frame sequence $DFS=\{DF^{1},DF^{2},\cdots ,DF^{m}\}$ of the first *m* frames with the three sequences constructs a four-stream representation of the corresponding action.

### 2.2. Extraction of Action Features

**Figure 2:**
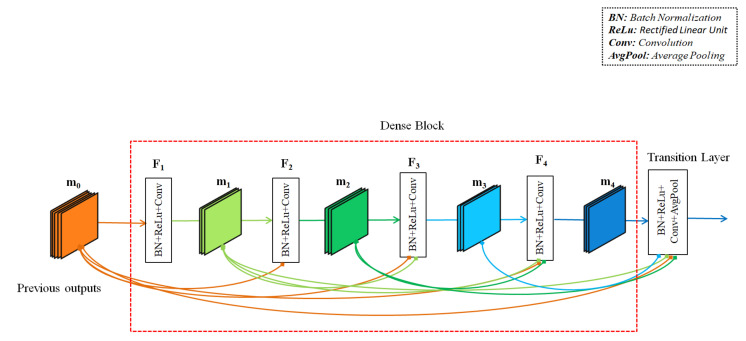
An example of a dense block in the DenseNet121 model.

To describe the action through frames of the four streams, the action features of temporal motion frames as well as depth frames are captured with the help of pre-trained 2D DenseNet121 [78] architecture. The DenseNet121 was trained on the popular ImageNet [79] image dataset. The DenseNet121 is known for alleviating the vanishing-gradient problem, strengthening feature propagation, encouraging feature reuse, and substantially reducing the number of parameters. The model consists of a single convolution layer with 64 filters of size 7×7 and a stride of 2, a single max pooling layer with a 3×3 max pooling sized filter and a stride of 2, four dense block layers, three transition layers, a single global average pooling layer of 7×7 sized filter and a single fully connected layer for classification. Every dense block has two repeated convolutions with two different sized filters of 1×1 and 3×3. The number of repetitions varies with the dense block layer. The 1×1 convolution layer is used as a bottleneck layer before each 3×3 convolution to improve the efficiency and speed of computations. In the dense block, the feature maps of all the previous layers are not summed but concatenated and used as current inputs. For example, if *k* is a current layer, then it receives all the output feature maps of previous layers, $m_{0},m_{2},\ldots ,m_{k-1}$ as input:

(4) $$m_{k}=F_{k}\left(\left[m_{0},m_{2},\ldots ,m_{k-1}\right]\right),$$

where $\left[m_{1},m_{2},\ldots ,m_{k-1}\right]$ is the concatenation of the outputs of previous layers (0,1,…,k−1) for an easy implementation in the current layer. In addition, $m_{k}$ is the output feature map of the current $k^{t}h$ layer. Here, $F_{k}\left(\cdot \right)$ is a composite function of batch normalization, ReLu and convolution operations on its inputs. Figure 2 represents an example dense block.

Each transition layer has a 1×1 convolution layer and a 2×2 average pooling layer with a stride of 2. The average pooling layer reduces the dimensionality of each feature map but retains the important information. The position of the transition layers is between two adjacent dense blocks to perform down-sampling (i.e., change the size of the feature maps) via convolution and pooling operations. Note that the batch normalization and ReLu mechanisms are used with each convolution layer in the dense block layers and in the subsequent transition layers.

**Figure 3:**
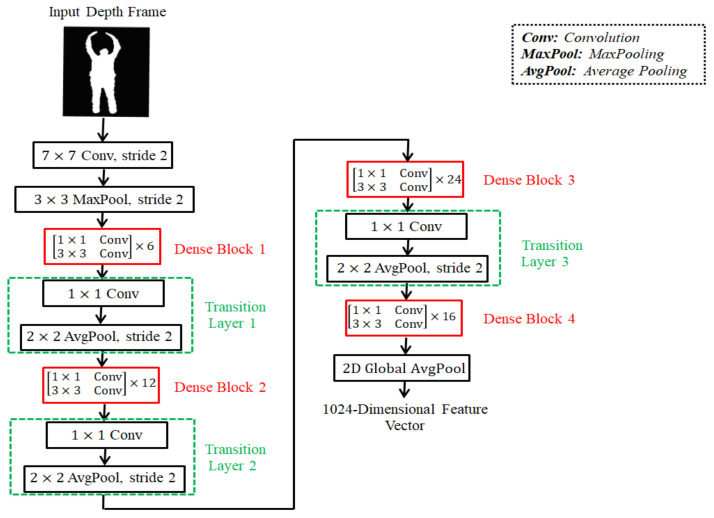
An example of feature extraction from depth frame using the DenseNet121 model.

The DenseNet121 architecture is employed here only to represent frames in terms of feature vectors individually rather than classifying those frames. The large ImageNet dataset covers all the classes of video classification problems, and thus, the ImageNet pre-trained weights of the DenseNet121 are reused in this work. In the model, a 2D global average pooling layer comes after the last convolution layer. The outcome of the 2D global average pooling layer is considered as the feature vector for representing the relevant frame. The global average pooling sums out the spatial information by accepting all the previous feature maps of the network. Figure 3 shows an example of feature extraction from a depth frame using the DenseNet121 model.

The implementation of DenseNet121 on each frame of the four streams outputs four sets of feature vectors. Each feature vector represents the frame in a space of 1024 dimensions.

### 2.3. Organization of Feature Vectors and Their Correlation Modeling

The input frames to the DenseNet121 model are basically members of a set of temporal data, i.e., the original orientation of frames along the temporal dimension. The model describes frames of a sequence through feature vectors individually. However, it cannot organize the feature vectors of frames along temporal dimension as a series with a single class label. Furthermore, a frame in the set can be strongly predicted by its previous frames because of the substantial correlations between the vectors of contiguous frames. Consequently, the vectors of contiguous frames are firmly correlated. The DenseNet121 descriptor also cannot do correlation modeling among the vectors. In this situation, the BLSTM [80] mechanism is adopted for arranging vectors along the temporal dimension and for modeling correlation among them.

The BLSTM is a combination of two unidirectional LSTMs [80]. One of the two LSTMs pushes input vectors from past to future (forward LSTM), whereas another LSTM runs them from future to past (backward LSTM). By concatenating the final two hidden states of the two LSTM cells, the output of BLSTM is computed. Because of the incorporation of a forward LSTM and backward LSTM outcomes, the information from both past and future at any point in time is preserved in BLSTM. To understand the working procedure of an LSTM, let $X=x_{0},x_{1},\cdots ,x_{S}$ be a set of *S* feature vectors (outputs of DenseNet121) of *D* dimensions representing depth frames. If $x_{t}\in X$ is input of an LSTM cell, then the final hidden state $h_{t}$ and the final cell state $c_{t}$ of the LSTM are computed as

(5) $$i_{t}=\sigma \left(w_{i}\cdot \left[h_{t-1},x_{t}\right]+b_{i}\right),$$

(6) $$\tilde{c_{t}}=tanh\left(w_{c}\cdot \left[h_{t-1},x_{t}\right]+b_{c}\right),$$

(7) $$f_{t}=\sigma \left(w_{f}\cdot \left[h_{t-1},x_{t}\right]+b_{f}\right),$$

(8) $$c_{t}=f_{t}⊙c_{t-1}+i_{t}⊙\tilde{c_{t}},$$

(9) $$o_{t}=\sigma \left(w_{o}\cdot \left[h_{t-1},x_{t}\right]+b_{o}\right),$$

(10) $$h_{t}=o_{t}⊙tanh\left(c_{t}\right),$$

where $h_{t}$ is the hidden state or final output of the LSTM cell at timestamp *t*. The $i_{t}$, $\tilde{c_{t}}$, $f_{t}$, and $o_{t}$ are outcomes of input (i), forget (f), and output (o) gates at the current timestamp *t* with weights $w_{i}$, $w_{c}$, $w_{f}$, and $w_{o}$ respectively. The symbol σ is the logistic sigmoid function, ⊙ is used for element-wise multiplication and tanh is a hyperbolic tangent function. In BLSTM, two hidden states $h_{t}^{forward}$ and $h_{t}^{backward}$ are computed using Equations (5)–(10) across the backward and forward LSTM cells a timestamp *t* against the input $x_{t}$. The final representation of $x_{t}$ is then calculated by the concatenation of $h_{t}^{forward}$ and $h_{t}^{backward}$ as

(11) $$h_{t}=\left[h_{t}^{forward},h_{t}^{backward}\right]$$

By using Equations (5)–(11), the output of the BLSTM concerning sequence $X\in R^{S\times D}$ is again a sequence $Y=(h_{1},h_{2},\cdots ,h_{S})$ of vectors of a specific length (equal to the output space of the BLSTM).

However, four sets of feature vectors, obtained from the utilization of DenseNet121 on the four streams, are fed into four different BLSTM cells separately. Each BLSTM converts the set to sequence by organizing the vectors along temporal dimension and modeling correlation among them. As a result, four BLSTM models output four different sequences corresponding to the four input sets of feature vectors.

### 2.4. Weight Assignment to Prominent Feature Vectors

Not all the frames in a sequence carry significant information. A number of the frames have more discriminating information than others. Therefore, there are many existing methods (e.g., the method in [29]) which selected the most informative frames of a sequence to describe the sequence. Unlike frame selection methods, our system gives special attention to the richer frames by weighting corresponding feature vectors. To do this, the MHSA algorithm [81] is employed on the set of feature vectors. The self-attention algorithm basically discovers those input vectors which are tremendously correlated with the remaining vectors. The algorithm labels these vectors as distinguished by multiplying them with weights. For its facile implementation commentary, assume the output of the previous BLSTM layer concerning sequence *X* is a sequence of *S* vectors of length $D^{\prime }$, i.e., $Y=\left(h_{1},h_{2},\cdots ,h_{S}\right)\in R^{S\times D^{\prime }}$. Each member vector of *Y* can be decomposed into several vectors of equal dimensions ($<D^{\prime }$). In fact, the vector decomposition yields a couple of sub-sequences ${\left\{Y_{i}\right\}}_{i=1}^{N}$ of sequence *Y* with properties $Y=\cup_{i=1}^{N}Y_{i}$ and $Y_{a}\cap Y_{b}=\emptyset$. Here, an individual $Y_{i}$ is another sequence of *S* vectors of length $d_{i}=\frac{D^{\prime }}{N}<D^{\prime }$. However, a sub-sequence $Y_{i}$ is represented using three different ways with three matrices $W_{i}^{Q}\in R^{d_{i}\times d_{q}^{i}}$, $W_{i}^{K}\in R^{d_{i}\times d_{k}^{i}}$, and $W_{i}^{V}\in R^{d_{i}\times d_{i}^{v}}$ as $Q_{i}\left(query\right)=Y_{i}W_{i}^{Q}$, $K_{i}\left(key\right)=Y_{i}W_{i}^{K}$, and $V_{i}\left(value\right)=Y_{i}W_{i}^{V}$ with $d_{i}^{k}=d_{i}^{q}$. The self-attention heads can be calculated simultaneously on all $Y_{i}$ with $Q_{i}=Y_{i}W_{i}^{Q}$, $K_{i}=Y_{i}W_{i}^{K}$, $V_{i}=Y_{i}W_{i}^{V}$ by

(12) $$H_{i}=A_{i}\left(Q_{i},K_{i},V_{i}\right)=softmax\left(\frac{Q_{i}K_{i}^{T}}{\sqrt{d_{i}^{k}}}\right)V_{i},$$

All the attention heads $H_{i}$ are concatenated to obtain the MHSA of sequence *Y* as

(13) $$MHSA\left(Y,Y\right)=Concat\left(H_{1},H_{2},\cdots ,H_{N}\right)W^{0}$$

The MHSA(Y,Y) is the new representation of sequence *Y* where the potential vectors are emphasized to boost the system and to play a key role in classification.

The output of four BLSTM layers are further passed to four different MHSA layers. The MHSA outputs are also four different sequences of vectors, but the most discriminating vectors are weighted.

### 2.5. Action Class Assignment

**Figure 4:**
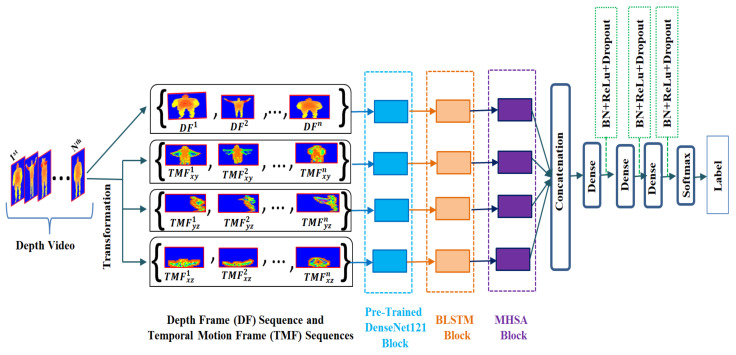
Our depth action classification system.

The outcomes of four MHSA layers are flattened independently and concatenated by an end-to-end procedure. Three dense layers are added to the concatenated output with three batch normalization (BN), dropout and rectified linear unit (ReLu) activation layers. The dropout is used to reduce data overfitting, and the BN is used to speed up the training process as well as make the training more stable. To predict the class index of the original input action video/depth sequence, another fully connected layer with softmax activation is considered where the dimensionality of the output space is the number of video classes. The entire architecture of our action classification task is shown in Figure 4.

## 3. Experiment and Results

The proposed framework is implemented in the Python Keras (TensorFlow 2.6.0) library on the Windows 10 platform. The computing hardware included an AMD Ryzen Threadripper 1900X 8-Core Processor of 3.9 GHz frequency, a memory of 64 GB, and an NVIDIA TITAN X (PASCAL) GPU. For evaluating our system, the precision, recall, F1-score and accuracy are taken into account as metrics. In addition, we performed ablation studies on the number of data streams as well as architectures. Experimental results are obtained on two benchmarks depth video datasets, i.e., MSRAction3D [82] and DHA [83].

### 3.1. Optimization of Hyper-Parameters

**Figure 5:**
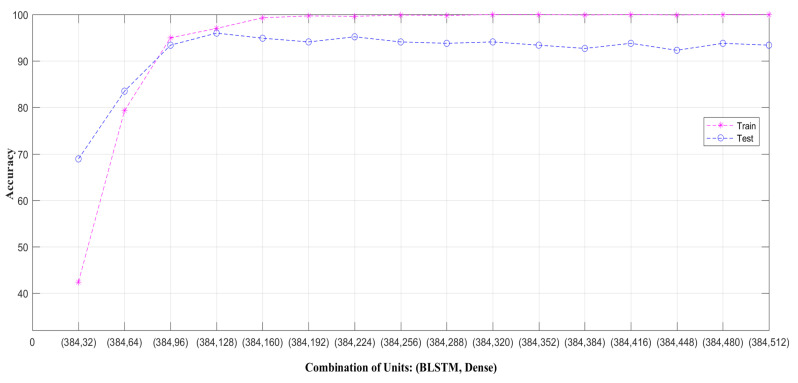
Setting of units on MSRAction3D in four BLSTM and three dense layers. Each result is regarding the combination (along the x-axis) of 384 BLSTM units and the number of dense layer units in the range of 32∼512.

In our work, the hyper-parameters are tuned for the BLSTM and MHSA blocks only, since the feature extraction is accomplished with the pre-trained 2D CNN deep model. In the feature extraction, each frame is resized to 224×224×3, and the 2D CNN is employed with the ImageNet pre-trained weights. However, for all the datasets, we experimentally set up the same values of all the hyper-parameters. The number of units (multiple of 32) in the four BLSTM and three dense layers are tuned in a range of 32∼512. The BLSTM and dense layer units are tuned for all the combinations of BLSTM and dense units as {(32, 32), (32, 128), …, (32, 512)}, {(64, 32), (64, 128), …, (64, 512)}, …, {(512, 32), (512, 128), …, (512, 512)}. In each combination, the first value is the BLSTM unit number, and the second value is the dense layer unit number. The optimal result is achieved with the combination of (384, 128) which is in the set {(384, 32), (384, 128), …, (384, 512)}. All the results of this set are shown in Figure 5. In the figure, the train and test accuracies are represented on the MSRAction3D dataset. Each result is about the combination (along x-axis) of the BLSTM unit number of 384 and a dense layer unit number in the range of 32∼512. Note that the training and test results are much closer at the combination of (384,128), and after that, the data overfitting is observed. Therefore, the BLSTM and dense layer unit numbers 384 and 128 are chosen for both datasets. The number of heads of MHSA is determined to be 2 from a set of values of {2,4,8,16} experimentally. The dropout rate of every dropout layer is tuned in 0∼0.8 in each dataset. The *categorical-crossentropy* loss function is employed for this multi-classification task. The *AdaMax* optimizer [84] is used to train our model on a batch size of 64 for 500 epochs. The learning rate of the optimizer is tuned in a range of 0.0001∼0.01 and set to 0.001 in all the experiments. The learning rate of 0.001 is also the default rate of the optimizer. The early stopping was not used here. Instead, we completed training for 500 epochs to obtain maximum insights into the training. While we could have trained for a much smaller number of epochs, we were interested in showing the training behavior for as many epochs as conveniently achievable. Moreover, the first sign of no improvement up to a specific number of epochs may not be the best time to stop training. This is because the model may become slightly worse before becoming much better; i.e., fluctuations may occur.

### 3.2. Evaluation on MSRAction3D Dataset

The MSRAction3D dataset [82] consists of 557 depth frame sequences (DFS) of 20 action classes. Those sequences were recorded by 10 persons performing different actions. The training sequences are recorded by persons of odd indices, whereas the test sequences are recorded by even indices actors. Since all other methods listed in Table 1 followed this data setup for MSRAction3D data, we retain a similar setup of training and test sets to assure fair comparison. There are 284 training DFS and 273 testing DFS in the dataset. After applying the data transformation on the training samples, in addition to DFS, there are 284 temporal motion frame sequences (TMFS) as training samples regarding each 2D plane, i.e., the number of $TMFS_{xy}$, $TMFS_{yz}$, and $TMFS_{xz}$ are 284 independently. Similarly, there are 273 samples for every testing data stream. Each sequence is split into several overlapping sub-sequences in training samples using step sizes of 3, 7, 10, and 13 (determined experimentally) of a fixed length. The length of each sub-sequence is fixed to 20 after conducting experiments on a set of lengths, i.e., {13,16,18,and 20}. After splitting all the training samples, there are 3807 training sequences of length 20 along each data stream. The strategy used on the training samples gives access to the entire sequence and also increases the number of training samples. Note that every sequence is processed to only 20 frames to propose a lightweight network. For more video frames, the networks have to be stacked deeper to obtain a larger temporal receptive field. Even though more frames could bring more information, they could also lead to noise issues.

**Figure 6:**
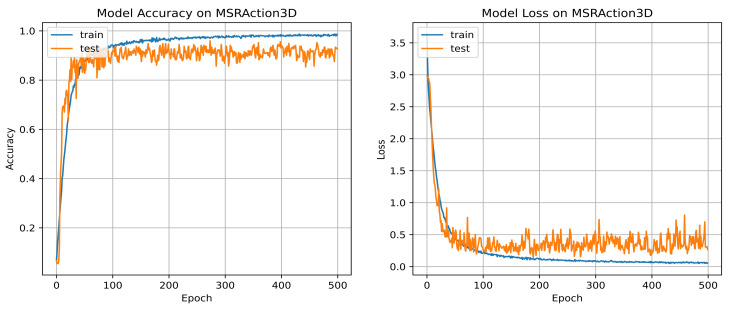
Accuracy and loss of our system for MSRAction3D dataset using *AdaMax* optimizer of 0.001 learning rate on 500 epochs.

In the testing data, the number of samples is kept unaltered (i.e., 273 samples), and a single sub-sequence of length 20 is trimmed out from a test sample when its length is more than 20. There are 20 timestamps for each BLSTM, since the sequence length is 20. Thus, there are 273 testing sequences along each data stream to validate our model. The three dropout rates are adjusted to 0.6, 0.65, and 0.65 for the MSRAction3D dataset. The accuracy and loss graphs over 500 epochs are shown in Figure 6.

**Figure 7:**
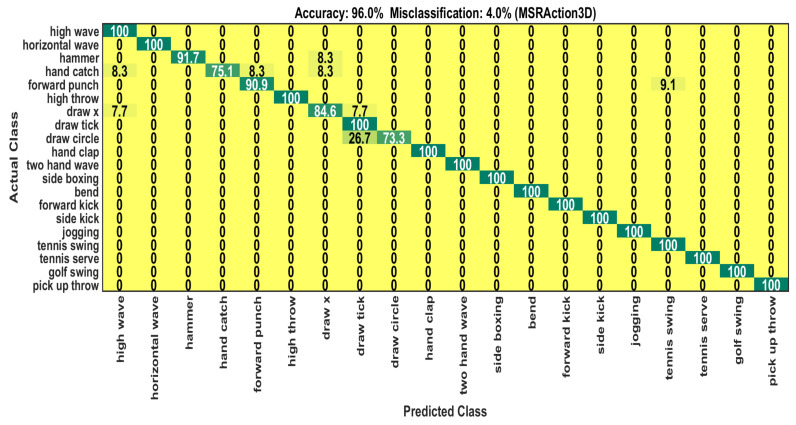
Confusion matrix on MSRAction3D test set.

The proposed system attains a significant recognition accuracy of 96% compared to other systems, as shown in Table 1. Our method outperforms the state-of-the-art 3D CNN depth action classification model [29] by 2.3%. The 3D CNN model used 16, 20, and 24 video frames, whereas we only utilized 20 video frames. The 3D CNN employed a couple of frame selection models to select potential frames which are not used in our method. The 3D CNN model averages the results obtained by the different numbers of frames and frame selection methods. Furthermore, our model has 26.98 million trainable parameters, which is a number that is much smaller than the 47.58 million parameters in the 3D CNN model. Our system can recognize 15 action classes with 100% accuracy out of 20 classes. However, the system exhibits errors in identifying the remaining 5 action classes. Because of the motion similarities of 5 action classes with other classes, the method is confused and cannot classify them correctly. For example, the system cannot achieve 100% recognition accuracy for action class *draw circle* since it suffers from 26.7% motion similarities with the *draw tick* class. Figure 7 shows the confusion in our system through a confusion matrix. In Table 1, the experimental result by changing the order of BLSTM and MHSA is also reported. The result shows that using the MHSA algorithm before the BLSTM results in an 18% fall in the accuracy. The comprehensive recognition performance is represented by Table 2.

### 3.3. Evaluation on DHA Dataset

**Figure 8:**
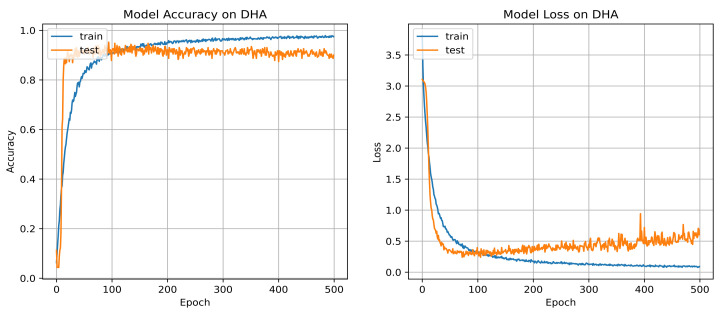
Accuracy and loss of our system for DHA dataset using *AdaMax* optimizer of 0.001 learning rate on 500 epochs.

**Figure 9:**
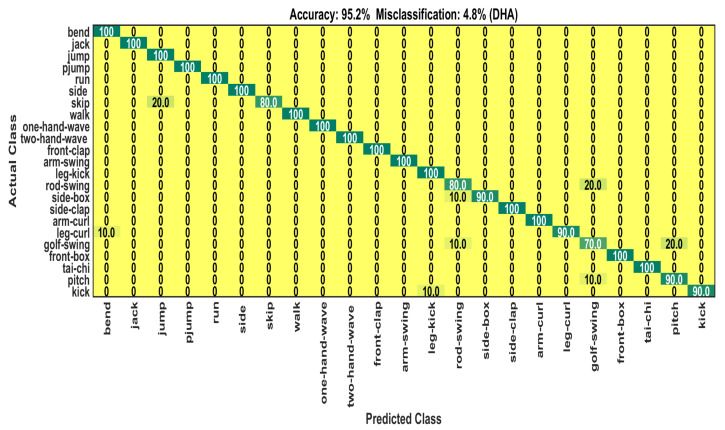
Confusion matrix on DHA test set.

The DHA dataset [82] has 483 depth frame sequences of 23 classes. In the dataset, every sequence is recorded by 21 performers. Similarly to other methods developed on this dataset, the sequences recorded by performers of odd indices (1, 3, 5, 7, and 9) are used as training data samples. The sequences recorded by performers of even indices (2, 4, 6, 8, and 10) are used as testing data samples. According to the setup, there are 253 samples for training and 230 samples for testing/validating the model. Using the same data splitting technique as used in the MSR-Action3D dataset, the training samples for each data stream are augmented from 253 to 3324 samples. The number of testing sequences is unchanged, i.e., 230. Like the previous dataset, every sequence in the training set and the testing set has a size of 20. There are 20 timestamps for each BLSTM, since the sequence length is 20. The three dropout rates are adjusted to 0.62, 0.65, and 0.65 as optimal for this dataset. The classification accuracy and loss graphs over 500 epochs are shown in Figure 8. Our system achieves overall 95.2% classification accuracy on the DHA test set (see Table 3). The table also shows the accuracy of implementing the MHSA algorithm before the BLSTM, and the accuracy is 4.3% lower than the accuracy of our proposed method. The observed accuracy is 100% for 16 action classes. The performance of the remaining seven classes reveals confusion with classes of similar motion cues (see Figure 9). Table 4 shows the classification performance of our system for each class extensively. The proposed approach outperforms other approaches significantly. Specifically, it had 2.3% greater accuracy than the recent 3D CNN-based deep learning recognition system, as reported in [29].

### 3.4. Ablation Study

This section evaluates the influence of the four data streams, the DenseNet121 model, and the order of BLSTM and MHSA mechanisms on the proposed method.

#### 3.4.1. Data Stream

Our system is built across the four-stream action data, i.e., a single depth frame sequence (DFS) and three temporal motion frame sequences (TMFS). We evaluate the significance of the four streams with respect to the single stream and the three streams. Therefore, we carry out experiments by replacing four streams with a single stream and three streams separately. The experimental results are shown in Table 5. In the table, the results based on the single, three, and four streams are reported for the two datasets. The four-stream model significantly outperforms the single-stream model. The three-stream model performs better than the single-stream model; however, it lags behind the four-stream model. The model using both the single stream and three streams, i.e., the four-stream model increases accuracy by 2.3% on MSRAction3D and 1.8% on DHA compared to using the three-stream model. The four-stream model increases accuracy by 31.2% on MSRAction3D and 13% on DHA compared to the single-stream model. The accuracy comparison between single-stream and four-stream models demonstrates the effectiveness of our approach for small-scale depth video datasets. More precisely, the MSR-Action3D and DHA datasets have 284 and 253 training samples, respectively. The training samples of both datasets are divided into overlapping samples with step sizes of 3, 7, 10 and 13. This allows an entire action video to be utilized in the training stage. Consequently, there are 3807 and 3324 training samples through the temporal video augmentation in MSR-Action3D and DHA, respectively. When we use these samples to train the single-stream model DFS+DenseNet121+BLSTM+MHSA, the recognition results are 64.8% and 82.2% on the MSR-Action3D and DHA, respectively. It is common for a deep model to perform poorly when the number of training samples is small. Due to this data deficit, it is imperative to convert the small-scale training data into large-scale training data. For making large-scale training data, the spatial video augmentation could be applied to the original training samples, since the temporal video augmentation is already applied. There are several types of spatial video augmentations, such as *Piece-wise Affine Transform*, *Super-pixel*, *Gaussian Blur*, *Invert Color*, *Random Rotate*, *Random Resize*, *Translate*, *Center Crop*, *Horizontal Flip*, *Vertical Flip*, *Add*, *Multiply*, *Downsamples*, *Upsamples*, *Elastic Transformation*, *Salt*, *Pepper*, *and Shear*. It should be noted that each type of augmentation is achieved through a frame-wise process. Moreover, selecting the perfect augmentation type for our model can be a challenge. Consequently, spatial video augmentation seems to be a complex implementation.

In order to avoid data augmentation (to preserve simplicity) and improve model performance without converting small-scale training data to large-scale, we propose that more three-stream networks of motion information can be added to the architecture with the single-stream network. The samples of these three streams contain motion information extracted from the samples of the single stream. So, these three streams have the same number of training samples as the single stream, so all four streams contain the same number of training samples. The four-stream model (DFS+TMFS)+DenseNet121+BLSTM+MHSA, processing four data streams (similar to ensemble of models) through four networks improves the accuracy from 64.8% to 96% on the MSRAction3D, and from 82.2% to 95.2% on the DHA, which is a significant improvement. Instead of the single-stream model, the proposed four-stream model increases the accuracy by 31.2% on the MSA-action3D dataset and by 13% on the DHA dataset. In summary, to improve the model performance, an additional three streams of motion data are included in the existing model instead of increasing training samples in the single stream. In these three streams, all the samples are obtained through processing the training samples of the single stream. The four-stream model works well when there are few training samples in the depth dataset because it increases accuracy by using small-scale training data. In fact, the motion information of the three streams helps to improve the recognition accuracy. Hence, the four-stream paradigm could be used instead of data augmentation when the depth dataset is small.

#### 3.4.2. Architecture

In addition to the DenseNet121 model, DenseNet169 and ResNet101V2 pre-trained models are employed to extract frame features. The other two models use more features to represent each frame. More specifically, the dimension of the DenseNet121 feature vector is 1024, whereas the dimension of the DenseNet169 feature vector is 1664 and the dimension of the ResNet101V2 feature vector is 2048. The experimental outcomes using the three models are illustrated in Table 6. DenseNet121 achieves the best accuracy among the three models on both datasets, although it uses fewer features than the other two models.

## 4. Conclusions

In this paper, we have developed a four-stream deep model through a limited number of training samples for directly classifying depth frame sequences to achieve 3D action recognition. To describe a depth action more effectively, a depth frame sequence was transformed to produce sequences of three multi-view temporal motion frames that configured the four data streams. The action features were captured from depth frames and temporal motion frames employing a pre-trained 2D DenseNet121 model. With the DenseNet121 deep features, the sequence classification model was built using a combination of BLSTM, MHSA, and dense layers. Our method was evaluated on two public small-scale depth datasets. The method achieved superiority over the existing deep learning methods as well as handcrafted methods significantly. In addition, the proposed deep sequence learning model, the four-stream model, was compared to the single-stream model (which uses depth frame sequences) and the three-stream model (which uses motion frame sequences). The performance of the four-stream model was superior to the single-stream and three-stream models when training samples were insufficient. In fact, the four-stream paradigm replaced data augmentation for a small dataset successfully. In addition to the DenseNet121 model, the DenseNet169 and ResNet101V2 were employed for the feature extraction task in the four-stream model, but their performance could not surpass the performance attained on DenseNet121. Furthermore, the order of the BLSTM and MHSA models was altered. Implementing the MHSA after the BLSTM helped build the promising sequence learning model. We have actually emphasized the development of a sequence learning method rather than increasing accuracy by large margins when the number of training samples is small. There are a number of alternatives that are not used in the method to improve accuracy: for example, spatial data augmentation, temporal data augmentation with diverse frame numbers, and the use of different parameter tuning techniques in model training, etc. The architecture is actually very simple and lightweight, although it appears to be complicated due to four data streams. It is an effective sequence classification model compared to the 3D CNN model, since it outperforms the current depth action recognition 3D CNN model (published on 20 April 2022) by 1.9% for MSRAction3D and by 2.3% for DHA. To achieve the state-of-the-art results, it requires fewer video frames and 20.6 million less trainable parameters than the 3D CNN model. We believe our proposed methodology will help the research community in exploring and developing models for other small-scale depth dataset problems as an alternative to data augmentation.

## Figures and Tables

**Table 1:** Comparison of action recognition accuracy (%) with state-of-the-art frameworks on the MSRAction3D test set.

Approach	Accuracy (%)
Decision-level-Fusion (MV) [49]	91.9
DMM-GLAC-FF [50]	89.38
DMM-GLAC-DF [50]	92.31
DMM-LBP-FF [51]	91.9
DMM-LBP-DF [51]	93.0
MTDMM [46]	95.97
CDF [48]	80.8
Skeleton-MSH [52]	90.98
3D HoT_S [53]	91.9
3D HoT_M [53]	88.3
SSTKDes [47]	95.60
Depth-STACOG [54]	75.82
DMM-GLAC [54]	89.38
WDMM [55]	90.0
DMM-UDTCWT [56]	92.67
3D CNN+DMM-Pyramid [64]	86.08
$3D^{2}$ CNN [70]	84.07
2D CNN+DMM-Pyramid [64]	91.21
Depth+1D CNN [31]	90.18
Multi-channel-CNN-Ensemble+Bag [35]	94.55
1D CNN+DTW [36]	95.6
3D CNN+DHI+Relieff+SVM [66]	92.8
Depth+3D CNN [29]	94.1
(DFS+TMFS)+DenseNet121+MHSA+BLSTM	78
**(DFS+TMFS)+DenseNet121+BLSTM+MHSA (Ours)**	**96**

**Table 2:** Class-specific classification report on MSRAction3D test set.

Class	Precision	Recall	F1-Score	Accuracy (%)	Confusion (%)
High wave	86.0	100	92.0	**100**	No
Horizontal wave	100	100	100	**100**	No
Hammer	100	92.0	96.0	91.7	Draw x (8.3)
Hand catch	100	75.0	86.0	75.1	High wave (8.3), Forward punch (8.3), Draw x (8.3)
Forward punch	91.0	91.0	91.0	90.9	Tennis swing (9.1)
High throw	100	100	100	**100**	No
Draw x	85.0	85.0	85.0	84.6	High wave (7.7), Draw tick (7.7)
Draw tick	75.0	100	86.0	**100**	No
Draw circle	100	73.0	85.0	73.3	Draw tick (26.7)
Hand clap	100	100	100	**100**	No
Two hand wave	100	100	100	**100**	No
Side boxing	100	100	100	**100**	No
Bend	100	100	100	**100**	No
Forward kick	100	100	100	**100**	No
Side kick	100	100	100	**100**	No
Jogging	100	100	100	**100**	No
Tennis swing	94.0	100	97.0	**100**	No
Tennis serve	100	100	100	**100**	No
Golf swing	100	100	100	**100**	No
Pick up and throw	100	100	100	**100**	No

**Table 3:** Comparison of our highest action recognition accuracy (%) with state-of-the-art frameworks on the DHA test set.

Approach	Accuracy (%)
SDM-BSM [57]	89.50
GTI-BoVW [58]	91.92
Depth WDMM [55]	81.05
RGB-VCDN [59]	84.32
VCDN [59]	88.72
Binary Silhouette [60]	91.97
DMM-UDTCWT [56]	94.2
Stridden DMM-UDTCWT [56]	94.6
VCA [61]	89.31
CAM [62]	87.24
Depth+3D CNN [29]	92.9
(DFS+TMFS)+DenseNet121+MHSA+BLSTM	90.9
**(DFS+TMFS)+DenseNet121+BLSTM+MHSA (Ours)**	**95.2**

**Table 4:** Class-specific comprehensive classification report on DHA test set.

Class	Precision	Recall	F1-Score	Accuracy (%)	Confusion (%)
Bend	91.0	100	95.0	**100**	No
Jack	100	100	100	**100**	No
Jump	83.0	100	91.0	**100**	No
Pjump	100	100	100	**100**	No
Run	100	100	100	**100**	No
Side	100	100	100	**100**	No
Skip	100	80.0	89.0	80.0	Jump (20.0)
Walk	100	100	100	**100**	No
One-hand-wave	100	100	100	**100**	No
Two-hand-wave	100	100	100	**100**	No
Front-clap	100	100	100	**100**	No
Arm-swing	100	100	100	**100**	No
Leg-kick	91.0	100	95.0	**100**	No
Rod-swing	80.0	80.0	80.0	80.0	Golf-swing (20.0)
Side-box	100	90.0	95.0	90.0	Rod-swing (10.0)
Side-clap	100	100	100	**100**	No
Arm-curl	100	100	100	**100**	No
Leg-curl	100	90.0	95.0	90.0	Bend (10.0)
Golf-swing	70.0	70.0	70.0	70.0	Rod-swing (10.0), Pitch (20.0)
Front-box	100	100	100	**100**	No
Tai-chi	100	100	100	**100**	No
Pitch	82.0	90.0	86.0	90.0	Golf-swing (10.0)
Kick	100	90.0	95.0	90.0	Leg-kick (10.0)

**Table 5:** Observation of effectiveness of four data streams in model development.

Approach	MSRAction3D Test Set	DHA Test Set
Single-stream: DFS+DenseNet121+BLSTM+MHSA	64.8	82.2
Three-stream: TMFS+DenseNet121+BLSTM+MHSA	93.7	93.4
**Four-stream: (DFS+TMFS)+DenseNet121+BLSTM+MHSA**	**96**	**95.2**

**Table 6:** Performance of different architectures using four data streams.

Approach	MSRAction3D Test Set	DHA Test Set
(DFS+TMFS)+ResNet101V2+BLSTM+MHSA	89.3	91.3
(DFS+TMFS)+DenseNet169+BLSTM+MHSA	93.4	93.9
**(DFS+TMFS)+DenseNet121+BLSTM+MHSA**	**96**	**95.2**

## Data Availability

Not applicable.
